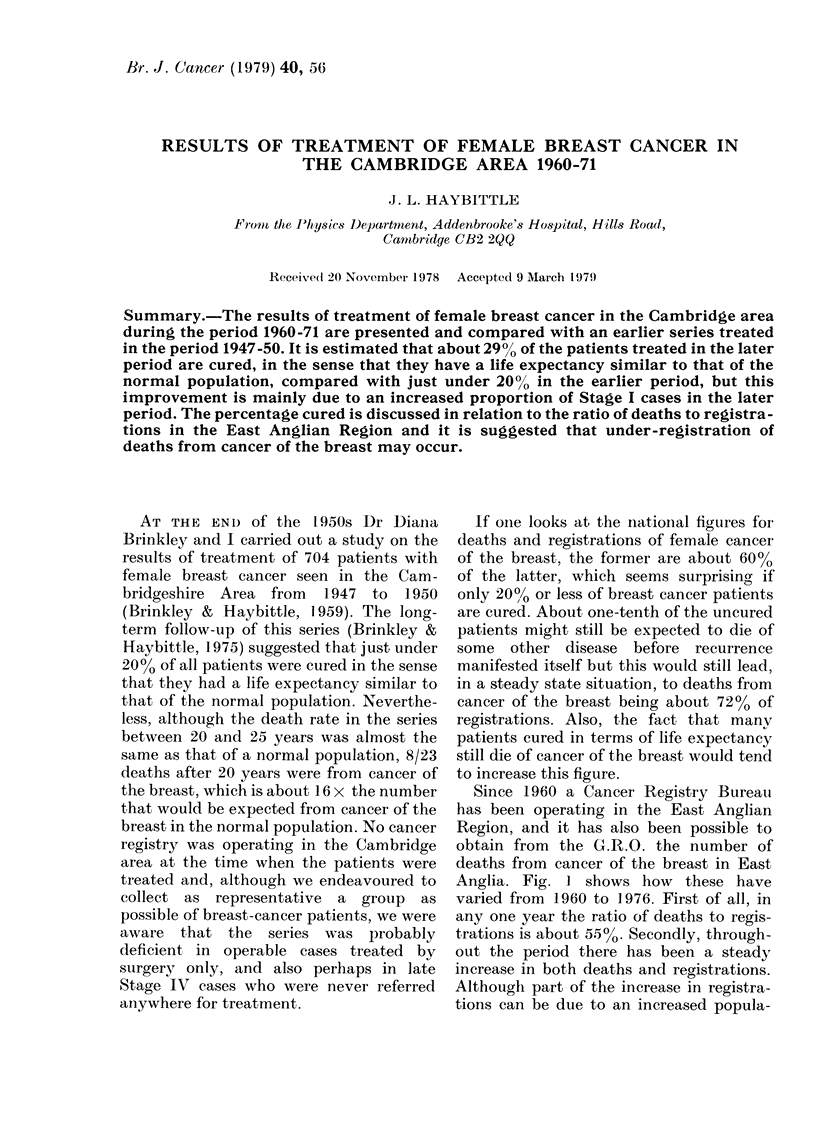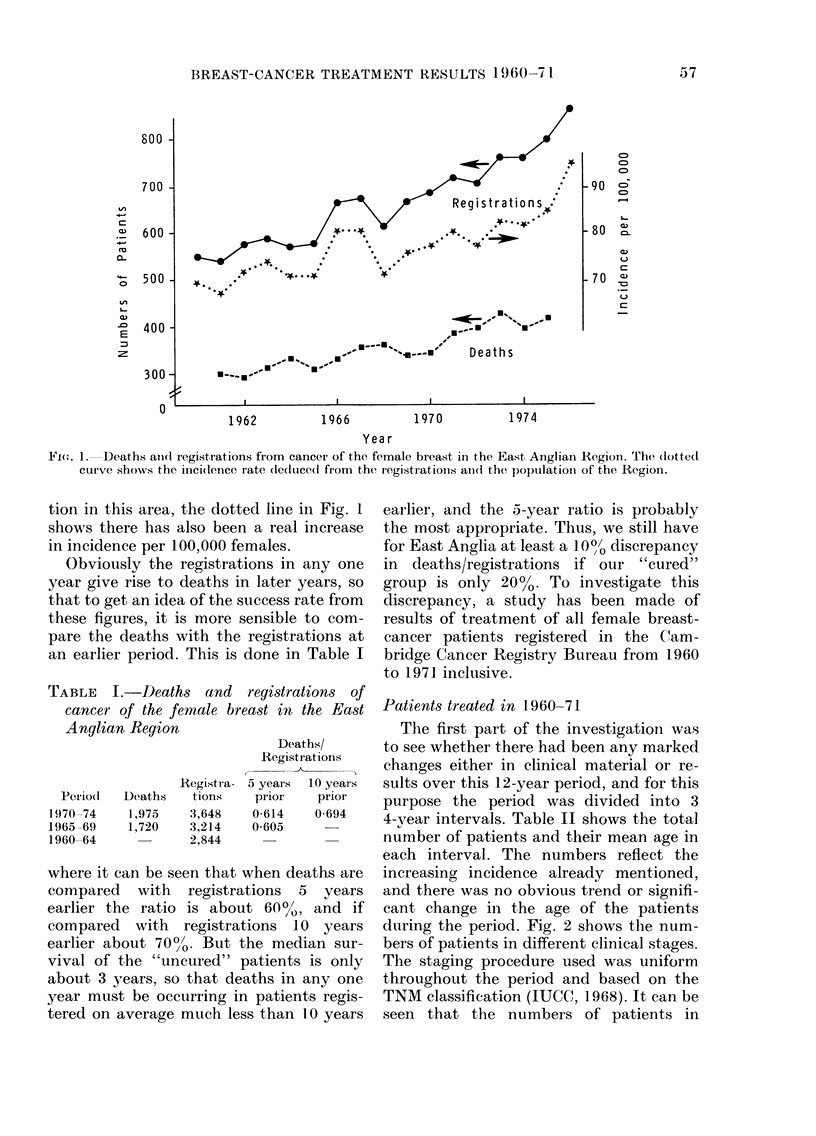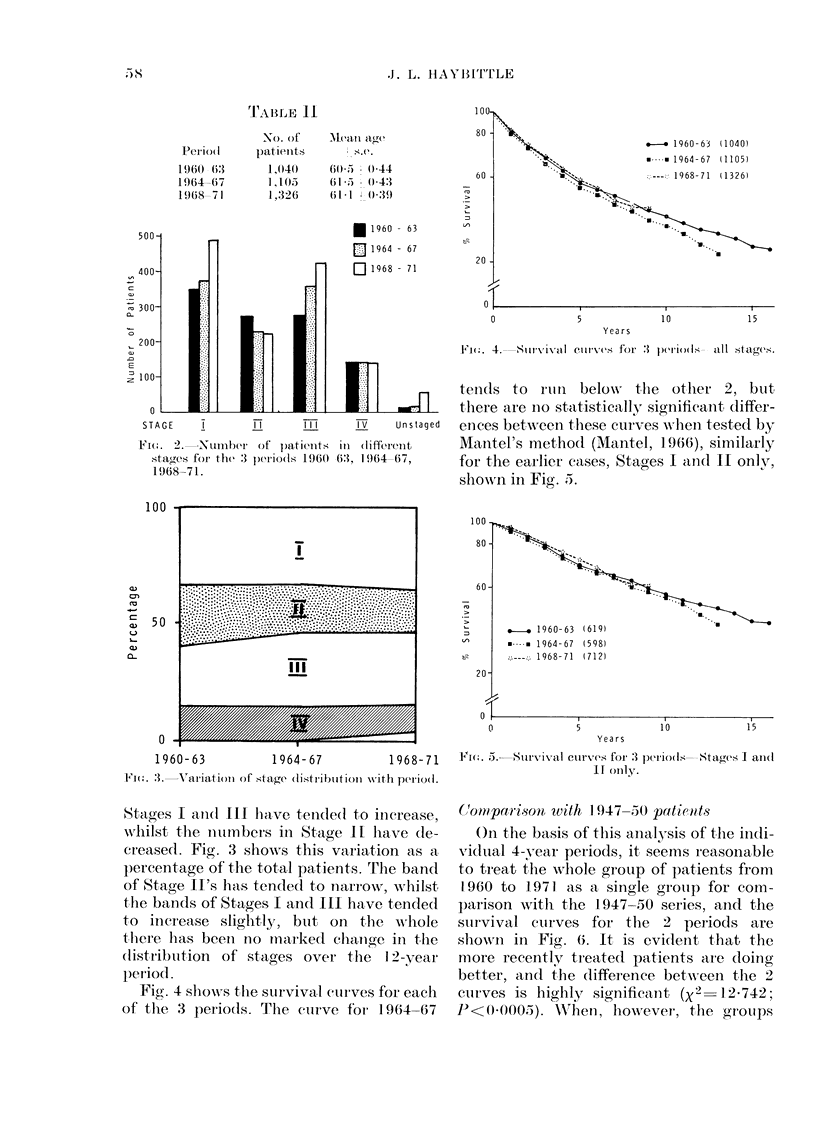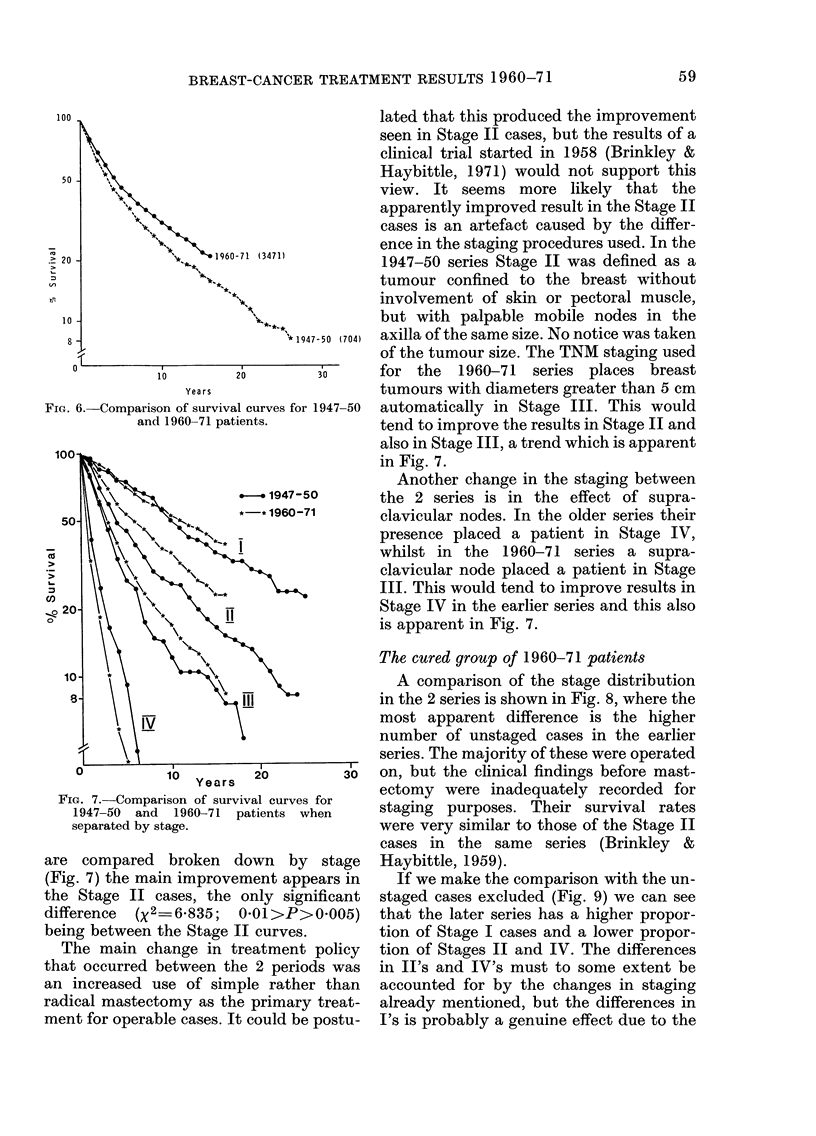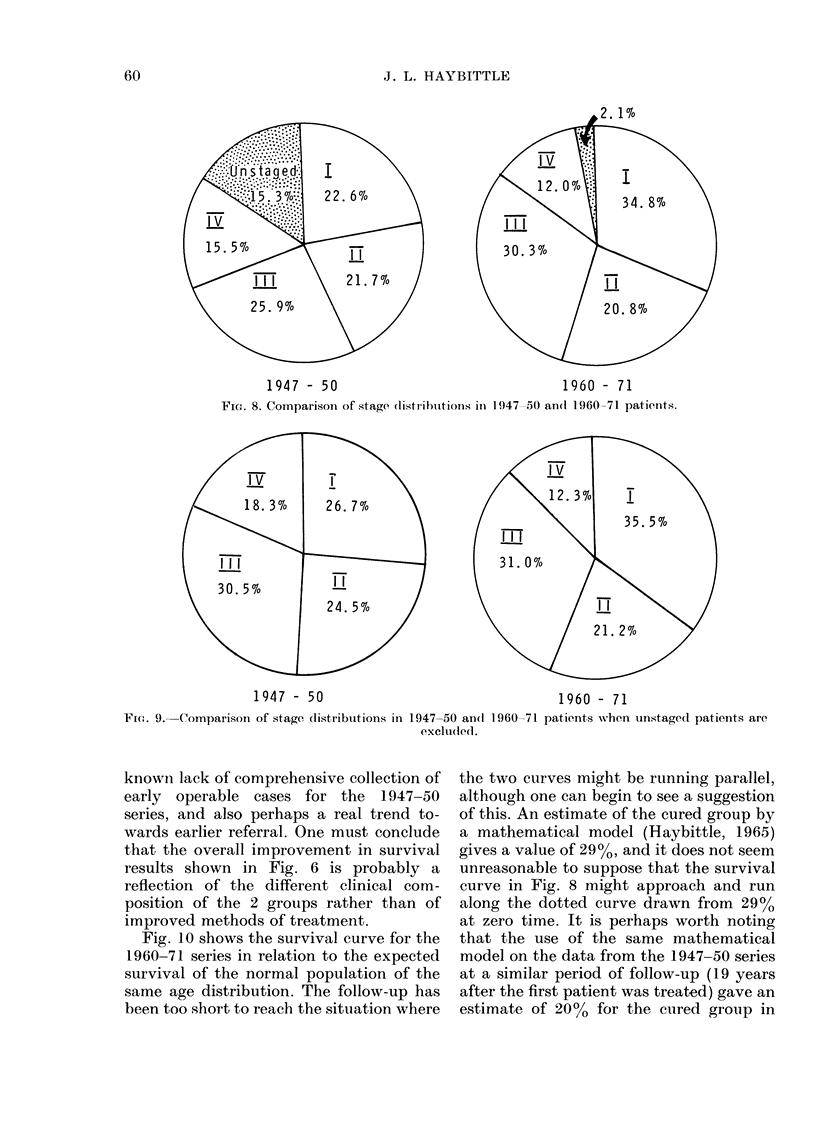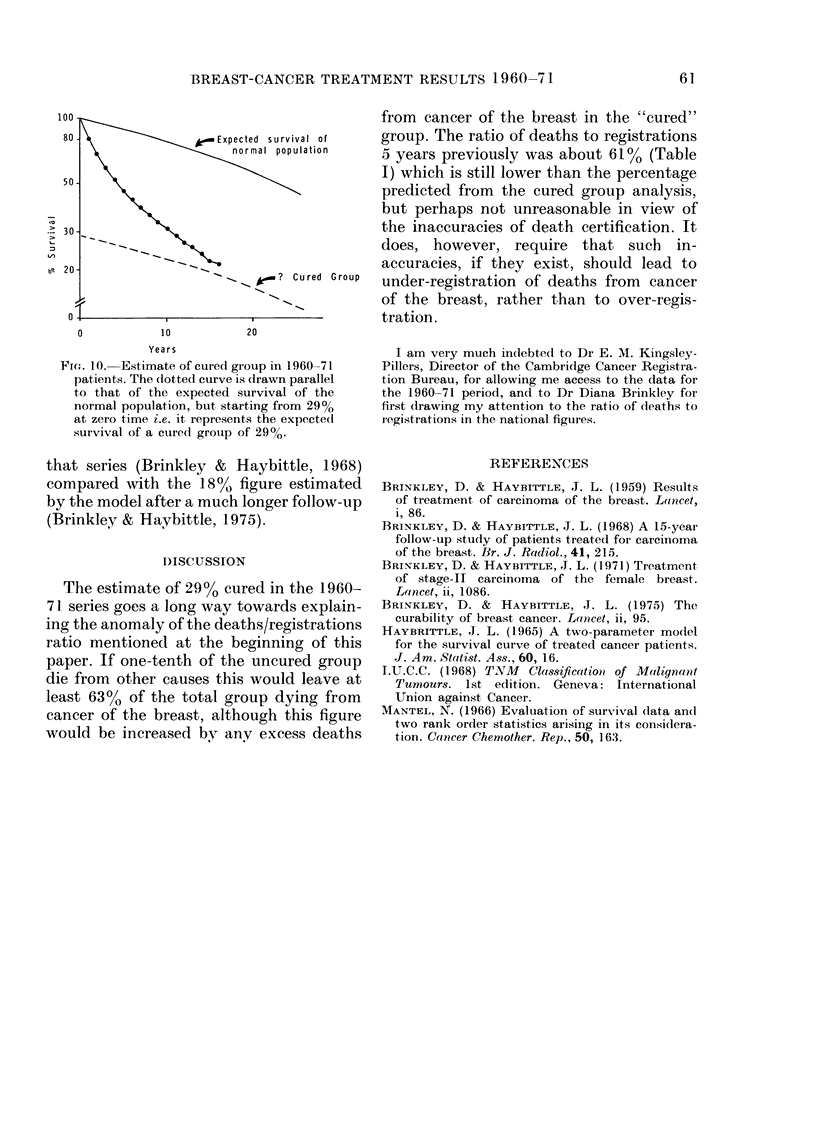# Results of treatment of female breast cancer in the Cambridge area 1960-71.

**DOI:** 10.1038/bjc.1979.140

**Published:** 1979-07

**Authors:** J. L. Haybittle

## Abstract

The results of treatment of female breast cancer in the Cambridge area during the period of 1960-71 are presented and compared with an earlier series treated in the period of 1947-50. It is estimated that about 29% of the patients treated in the later period are cured, in the sense that they have a life expectancy similar to that of the normal population, compared with just under 20% in the earlier period, but this improvement is mainly due to an increased proportion of Stage I cases in the latter period. The percentage cured is discussed in relation to the ratio of deaths to registrations in the East Anglian Region and it is suggested that under-registration of deaths from cancer of the breast may occur.


*Br. J. Cancer* (1979) **40**, 56

### RESULTS OF TREATMENT OF FEMALE BREAST CANCER IN THE CAMBRIDGE AREA 1960-71

J. L. HAYBITTLE

*From the Physics Department, Addenbrooke's Hospital, Hills Road, Cambridge CB2 2QQ*

Received 20 November 1978 Accepted 9 March 1979

**Summary.—The results of treatment of female breast cancer in the Cambridge area during the period 1960-71 are presented and compared with an earlier series treated in the period 1947-50. It is estimated that about 29% of the patients treated in the later period are cured, in the sense that they have a life expectancy similar to that of the normal population, compared with just under 20% in the earlier period, but this improvement is mainly due to an increased proportion of Stage I cases in the later period. The percentage cured is discussed in relation to the ratio of deaths to registrations in the East Anglian Region and it is suggested that under-registration of deaths from cancer of the breast may occur.**

AT THE END of the 1950s Dr Diana Brinkley and I carried out a study on the results of treatment of 704 patients with female breast cancer seen in the Cambridgeshire Area from 1947 to 1950 (Brinkley & Haybittle, 1959). The long-term follow-up of this series (Brinkley & Haybittle, 1975) suggested that just under 20% of all patients were cured in the sense that they had a life expectancy similar to that of the normal population. Nevertheless, although the death rate in the series between 20 and 25 years was almost the same as that of a normal population, 8/23 deaths after 20 years were from cancer of the breast, which is about 16× the number that would be expected from cancer of the breast in the normal population. No cancer registry was operating in the Cambridge area at the time when the patients were treated and, although we endeavoured to collect as representative a group as possible of breast-cancer patients, we were aware that the series was probably deficient in operable cases treated by surgery only, and also perhaps in late Stage IV cases who were never referred anywhere for treatment.

If one looks at the national figures for deaths and registrations of female cancer of the breast, the former are about 60% of the latter, which seems surprising if only 20% or less of breast cancer patients are cured. About one-tenth of the uncured patients might still be expected to die of some other disease before recurrence manifested itself but this would still lead, in a steady state situation, to deaths from cancer of the breast being about 72% of registrations. Also, the fact that many patients cured in terms of life expectancy still die of cancer of the breast would tend to increase this figure.

Since 1960 a Cancer Registry Bureau has been operating in the East Anglian Region, and it has also been possible to obtain from the G.R.O. the number of deaths from cancer of the breast in East Anglia. Fig. 1 shows how these have varied from 1960 to 1976. First of all, in any one year the ratio of deaths to registrations is about 55%. Secondly, throughout the period there has been a steady increase in both deaths and registrations. Although part of the increase in registrations can be due to an increased popula-

FIG. 1.—Deaths and registrations from cancer of the female breast in the East Anglian Region. The dotted curve shows the incidence rate deduced from the registrations and the population of the Region.

tion in this area, the dotted line in Fig. 1 shows there has also been a real increase in incidence per 100,000 females.

Obviously the registrations in any one year give rise to deaths in later years, so that to get an idea of the success rate from these figures, it is more sensible to compare the deaths with the registrations at an earlier period. This is done in Table I

TABLE I.—*Deaths and registrations of cancer of the female breast in the East Anglian Region*

| | | | Deaths/ Registrations | |
|---|---|---|---|---|
| Period | Deaths | Registrations | 5 years prior | 10 years prior |
| 1970–74 | 1,975 | 3,648 | 0·614 | 0·694 |
| 1965–69 | 1,720 | 3,214 | 0·605 | — |
| 1960–64 | — | 2,844 | — | — |

where it can be seen that when deaths are compared with registrations 5 years earlier the ratio is about 60%, and if compared with registrations 10 years earlier about 70%. But the median survival of the "uncured" patients is only about 3 years, so that deaths in any one year must be occurring in patients registered on average much less than 10 years earlier, and the 5-year ratio is probably the most appropriate. Thus, we still have for East Anglia at least a 10% discrepancy in deaths/registrations if our "cured" group is only 20%. To investigate this discrepancy, a study has been made of results of treatment of all female breast-cancer patients registered in the Cambridge Cancer Registry Bureau from 1960 to 1971 inclusive.

*Patients treated in* 1960–71

The first part of the investigation was to see whether there had been any marked changes either in clinical material or results over this 12-year period, and for this purpose the period was divided into 3 4-year intervals. Table II shows the total number of patients and their mean age in each interval. The numbers reflect the increasing incidence already mentioned, and there was no obvious trend or significant change in the age of the patients during the period. Fig. 2 shows the numbers of patients in different clinical stages. The staging procedure used was uniform throughout the period and based on the TNM classification (IUCC, 1968). It can be seen that the numbers of patients in

TABLE II

| Period | No. of patients | Mean age ± s.e. |
|---|---|---|
| 1960–63 | 1,040 | 60·5 ± 0·44 |
| 1964–67 | 1,105 | 61·5 ± 0·43 |
| 1968–71 | 1,326 | 61·1 ± 0·39 |

FIG. 2.—Number of patients in different stages for the 3 periods 1960–63, 1964–67, 1968–71.

FIG. 3.—Variation of stage distribution with period.

Stages I and III have tended to increase, whilst the numbers in Stage II have decreased. Fig. 3 shows this variation as a percentage of the total patients. The band of Stage II's has tended to narrow, whilst the bands of Stages I and III have tended to increase slightly, but on the whole there has been no marked change in the distribution of stages over the 12-year period.

Fig. 4 shows the survival curves for each of the 3 periods. The curve for 1964–67

FIG. 4.—Survival curves for 3 periods—all stages.

tends to run below the other 2, but there are no statistically significant differences between these curves when tested by Mantel's method (Mantel, 1966), similarly for the earlier cases, Stages I and II only, shown in Fig. 5.

FIG. 5.—Survival curves for 3 periods—Stages I and II only.

*Comparison with 1947–50 patients*

On the basis of this analysis of the individual 4-year periods, it seems reasonable to treat the whole group of patients from 1960 to 1971 as a single group for comparison with the 1947–50 series, and the survival curves for the 2 periods are shown in Fig. 6. It is evident that the more recently treated patients are doing better, and the difference between the 2 curves is highly significant ($\chi^2 = 12{\cdot}742$; $P < 0{\cdot}0005$). When, however, the groups

FIG. 6.—Comparison of survival curves for 1947–50 and 1960–71 patients.

FIG. 7.—Comparison of survival curves for 1947–50 and 1960–71 patients when separated by stage.

are compared broken down by stage (Fig. 7) the main improvement appears in the Stage II cases, the only significant difference ($\chi^2 = 6{\cdot}835$; $0{\cdot}01 > P > 0{\cdot}005$) being between the Stage II curves.

The main change in treatment policy that occurred between the 2 periods was an increased use of simple rather than radical mastectomy as the primary treatment for operable cases. It could be postulated that this produced the improvement seen in Stage II cases, but the results of a clinical trial started in 1958 (Brinkley & Haybittle, 1971) would not support this view. It seems more likely that the apparently improved result in the Stage II cases is an artefact caused by the difference in the staging procedures used. In the 1947–50 series Stage II was defined as a tumour confined to the breast without involvement of skin or pectoral muscle, but with palpable mobile nodes in the axilla of the same size. No notice was taken of the tumour size. The TNM staging used for the 1960–71 series places breast tumours with diameters greater than 5 cm automatically in Stage III. This would tend to improve the results in Stage II and also in Stage III, a trend which is apparent in Fig. 7.

Another change in the staging between the 2 series is in the effect of supraclavicular nodes. In the older series their presence placed a patient in Stage IV, whilst in the 1960–71 series a supraclavicular node placed a patient in Stage III. This would tend to improve results in Stage IV in the earlier series and this also is apparent in Fig. 7.

### *The cured group of* 1960–71 *patients*

A comparison of the stage distribution in the 2 series is shown in Fig. 8, where the most apparent difference is the higher number of unstaged cases in the earlier series. The majority of these were operated on, but the clinical findings before mastectomy were inadequately recorded for staging purposes. Their survival rates were very similar to those of the Stage II cases in the same series (Brinkley & Haybittle, 1959).

If we make the comparison with the unstaged cases excluded (Fig. 9) we can see that the later series has a higher proportion of Stage I cases and a lower proportion of Stages II and IV. The differences in II's and IV's must to some extent be accounted for by the changes in staging already mentioned, but the differences in I's is probably a genuine effect due to the

FIG. 8. Comparison of stage distributions in 1947–50 and 1960–71 patients.

FIG. 9.—Comparison of stage distributions in 1947–50 and 1960–71 patients when unstaged patients are excluded.

known lack of comprehensive collection of early operable cases for the 1947–50 series, and also perhaps a real trend towards earlier referral. One must conclude that the overall improvement in survival results shown in Fig. 6 is probably a reflection of the different clinical composition of the 2 groups rather than of improved methods of treatment.

Fig. 10 shows the survival curve for the 1960–71 series in relation to the expected survival of the normal population of the same age distribution. The follow-up has been too short to reach the situation where the two curves might be running parallel, although one can begin to see a suggestion of this. An estimate of the cured group by a mathematical model (Haybittle, 1965) gives a value of 29%, and it does not seem unreasonable to suppose that the survival curve in Fig. 8 might approach and run along the dotted curve drawn from 29% at zero time. It is perhaps worth noting that the use of the same mathematical model on the data from the 1947–50 series at a similar period of follow-up (19 years after the first patient was treated) gave an estimate of 20% for the cured group in

FIG. 10.—Estimate of cured group in 1960–71 patients. The dotted curve is drawn parallel to that of the expected survival of the normal population, but starting from 29% at zero time *i.e.* it represents the expected survival of a cured group of 29%.

that series (Brinkley & Haybittle, 1968) compared with the 18% figure estimated by the model after a much longer follow-up (Brinkley & Haybittle, 1975).

### DISCUSSION

The estimate of 29% cured in the 1960–71 series goes a long way towards explaining the anomaly of the deaths/registrations ratio mentioned at the beginning of this paper. If one-tenth of the uncured group die from other causes this would leave at least 63% of the total group dying from cancer of the breast, although this figure would be increased by any excess deaths from cancer of the breast in the "cured" group. The ratio of deaths to registrations 5 years previously was about 61% (Table I) which is still lower than the percentage predicted from the cured group analysis, but perhaps not unreasonable in view of the inaccuracies of death certification. It does, however, require that such inaccuracies, if they exist, should lead to under-registration of deaths from cancer of the breast, rather than to over-registration.

I am very much indebted to Dr E. M. Kingsley-Pillers, Director of the Cambridge Cancer Registration Bureau, for allowing me access to the data for the 1960–71 period, and to Dr Diana Brinkley for first drawing my attention to the ratio of deaths to registrations in the national figures.

### REFERENCES

BRINKLEY, D. & HAYBITTLE, J. L. (1959) Results of treatment of carcinoma of the breast. *Lancet*, i, 86.

BRINKLEY, D. & HAYBITTLE, J. L. (1968) A 15-year follow-up study of patients treated for carcinoma of the breast. *Br. J. Radiol.*, **41**, 215.

BRINKLEY, D. & HAYBITTLE, J. L. (1971) Treatment of stage-II carcinoma of the female breast. *Lancet*, ii, 1086.

BRINKLEY, D. & HAYBITTLE, J. L. (1975) The curability of breast cancer. *Lancet*, ii, 95.

HAYBRITTLE, J. L. (1965) A two-parameter model for the survival curve of treated cancer patients. *J. Am. Statist. Ass.*, **60**, 16.

I.U.C.C. (1968) *TNM Classification of Malignant Tumours*. 1st edition. Geneva: International Union against Cancer.

MANTEL, N. (1966) Evaluation of survival data and two rank order statistics arising in its consideration. *Cancer Chemother. Rep.*, **50**, 163.